# Continuous-wave Y-band planar BWO with wide tunable bandwidth

**DOI:** 10.1038/s41598-017-18740-w

**Published:** 2018-01-10

**Authors:** Hongzhu Xi, Jianguo Wang, Zhaochang He, Gang Zhu, Yue Wang, Hao Wang, Zaigao Chen, Rong Li, Luwei Liu

**Affiliations:** 1 The Institute of Anhui Huadong photoelectric Technology, Wuhu, Anhui 241002 China; 2 Northwest Institute of Nuclear Technology, Xi’an, Shaanxi 710024 China; 3 Key Laboratory for Physical Electronics and Devices of Ministry of Education, Xi’an Jiaotong University, Xi’an, 710049 China

## Abstract

A high performance continuous-wave (CW) backward wave oscillator (BWO) with planar slow wave structure (SWS) and sheet electron beam in Y-band is presented in this paper. The mode selection is discussed by studying the dispersion curve of SWSs, distributions of the electric field, and particle-in-cell simulation results, showing that the designed BWO operates in the fundamental mode TM_11_. The planar SWSs are fabricated by using the UV-LIGA technology with the processing error less than 0.003 mm. The electron gun can provide the 2.5 mm × 0.14 mm sheet electron beam with maximum current density of 57 A/cm^2^ at the CW mode. Experimental results show that the developed BWO can operate in the fundamental mode TM_11_ and generate the state-of-art output power of 182 mW at the frequency of 0.3426 THz with a large frequency tuning range from 0.318 THz to 0.359 THz.

## Introduction

Radiation of the terahertz (THz) wave has drawn lots of attention in many areas of science and technology, including the plasma diagnostic in nuclear fusion, high data rate communications, remote high-resolution imaging, chemical spectroscopy, materials research, deep space research and communications, basic biological spectroscopy and biomedical diagnostics^1–8^. For example, to understand the critical phenomenon as the anomalous transport of the fusion plasma, the collective Thomson scattering at the terahertz frequency is studied^5,7^. The fusion plasma is radiated by the terahertz beam which is scattered by the charged particles, and the scattered signal is detected by an array of receivers. Then, the scattered signal is used to map out the location, wavenumber spectrum, and strength of the turbulence^7^. To perform the experiments of collective Thomson scattering, a THz radiation source with enough power will be needed, the vacuum electronic devices (VEDs) with high output power may be one of the suitable choices of the terahertz sources^9^.

Among the VEDs^9–25^, the free electron laser (FEL) and gyrotron can generate the terahertz wave of kilowatts in the terahertz band^9–12^, because of the large peripheral apparatus and high guiding magnetic field, these VEDs are usually used in some famous laboratories, such as Institute of Applied Physics, Russia, and Jefferson Lab, USA. The backward wave oscillator (BWO) is a promising candidate that can produce the CW terahertz waves with several hundreds of mW with the advantages of simple structure, small volume, light weight, room temperature operation, and wide-range tuning of the frequency^7,9,13–16^.

We developed a BWO-like CW clinotron with sheet electron beam of 5 kV at 0.26 THz^15^. The slow wave structures (SWSs) are the planar comb gratings fabricated by using the wire electrical discharge machining(WEDM) technology with the processing error less than 5 μm. Experimental results show that the developed clinotron can generate the output power of 820 mW at the frequency of 0.26 THz with a large frequency tuning range from 0.25 THz to 0.262 THz. There are some advantages of this kind of BWO. First, as the periodic length of the SWSs is only tens of μm, the electrons with high energy may destroy the SWSs, and hence, using the low voltage of 5 kV can guarantee the long life time of the device^14,15^. Now, the life time of our device at 0.26 THz reaches as high as about 1000 hours^15^. Second, using the low current density of 57 A/cm^2^ can decrease the requirement of the guiding magnetic field. Third, the electron beam without compression can reduce the size and weight of the device. And the fourth, compared with other types of SWS, the planar comb gratings are relatively simpler, so they are easier to manufacture and assemble^19–22^. For the terahertz wave above 0.3 THz, one type of “Clinotron-0.95” can work at 272–334 GHz with output power of 50–100 mW as reported^14^. To obtain the terahertz wave with the output power of watt-level or hundreds of mW at the frequency above 0.3 THz, many research works are being conducted, but most of them are still at the stages of theoretical design and numerical simulations^7^.

## Physical Design of the Planar BWO

**Figure 1:**
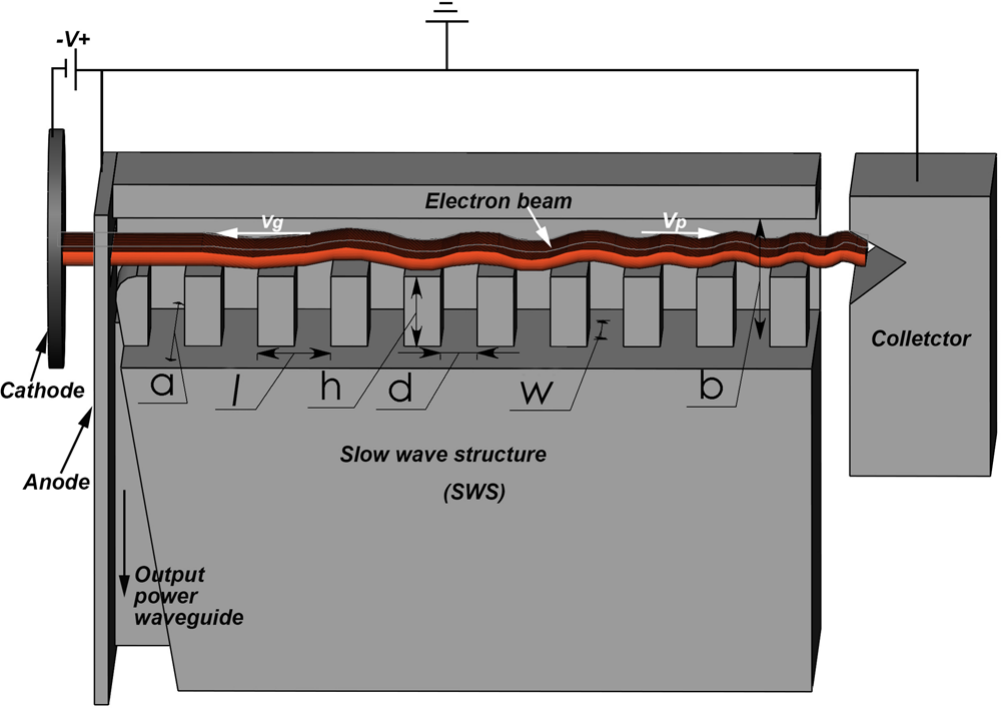
Schematic of the designed BWO. Width of waveguide (*a*), 7.2 mm; height of waveguide (*b*), 1.8 mm; period of grating (*l*), 0.1 mm; width of grating (*w*), 2.5 mm; height of slots (*h*), 0.16 mm; width of slots (*d*), 0.058 mm; and length of grating ($L$), 14 mm.

Figure 1 schematically shows the designed BWO, in which the planar periodic SWS supports the induced traveling electromagnetic wave interacting with the sheet electron beam. The synchronism condition of the BWO is located at the intersection point of the beam line of slope ${v}_{e}$ (the beam velocity) with the −1*st* order spatial harmonic among the spatial harmonic waves supported by the SWS. If the beam velocity *v*_*e*_ matches the forward phase velocity *v*_*p*_ of the wave, the electron beam will bunch and encounter synchronously the decelerating phase of the wave’s electric field ${E}_{z}$ between each gap in the SWS, leading to a net loss of energy in the beam and a growth of the wave. The generated wave’s energy travels backwards with the group velocity *v*_*g*_, and couples into the output waveguide.

Based on the parameters of the sheet electron gun and our processing conditions, the synchronism condition of the BWO is set as ${\rm{\phi }}$(=${k}_{z}l$) = 5π/3 for phase synchronism with the 5.0 keV electron beam. The period $l$ of 0.1 mm is derived from ${\rm{\phi }}$(=${k}_{z}l$) = $\frac{2\pi {f}_{0}}{{v}_{e}}l$, where *f*_0_ = 0.34 THz, and ${v}_{e}$ = 0.136 *c*, here *c* is the speed of light in the free space. The SWS is installed into a rectangular waveguide with cross section of *a* × *b*. After optimization of the other parameters of SWS, the geometric parameters are presented in Fig. 1.

**Figure 2:**
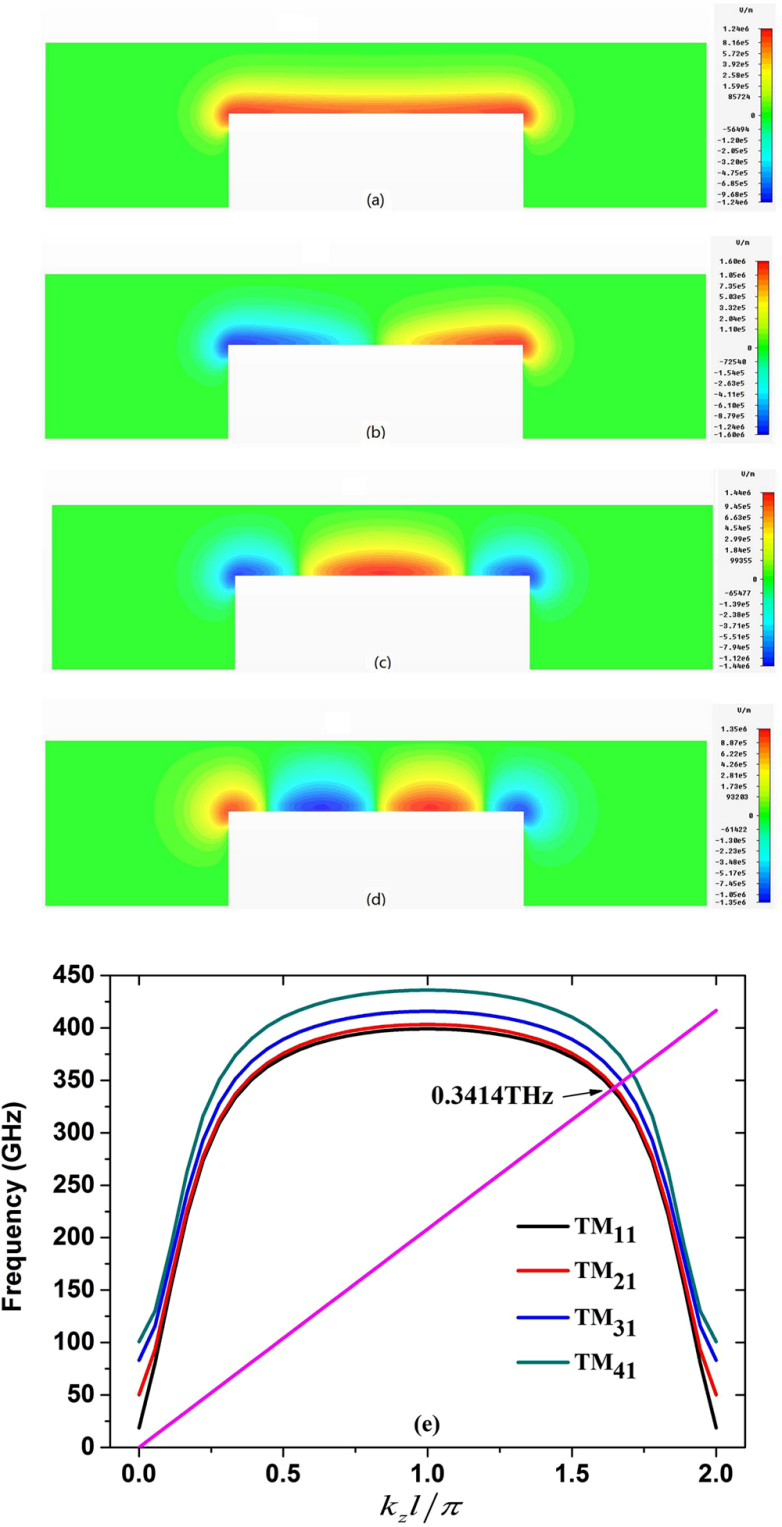
The lowest four modes inside the designed BWO and their dispersion curves. (**a**) TM_11_, (**b**) TM_21_, (**c**) TM_31_, (**d**) TM_41_, and (**e**) dispersion curves.

There exist many harmonic waves supported by the planar SWS, so it is crucial for successfully designing a VED in the terahertz range to select the proper electromagnetic mode and avoid the mode competition^26–28^. To do this, we calculate the four lowest modes and the dispersion curves by using the geometric parameters listed in Fig. 1, and depict them in Fig. 2. The antisymmetric TM_21_ and TM_41_ modes cannot be excited by the symmetric planar SWS and sheet electron beam. Next, let’s look at the field distributions of the other two symmetric TM_11_ and TM_31_ modes. First, for the TM_11_ mode, the electric field is a surface wave, decaying exponentially from the top surface of SWS to the up wall of waveguide^23–28^, but the position of strongest field is a little bit high above the top surface of SWS for the TM_31_ mode. Second, the coupling impedance of the TM_11_ mode is higher than that of TM_31_ mode. And hence, the terahertz wave with the TM_11_ mode is easier to be excited than the TM_31_ mode if the sheet electron beam is as closer as possible to the top surface of SWS. Figure 2(e) depicts the dispersion curves of the designed BWO, indicating that the working point is about 0.3414 THz for the TM_11_ mode.

**Figure 3:**
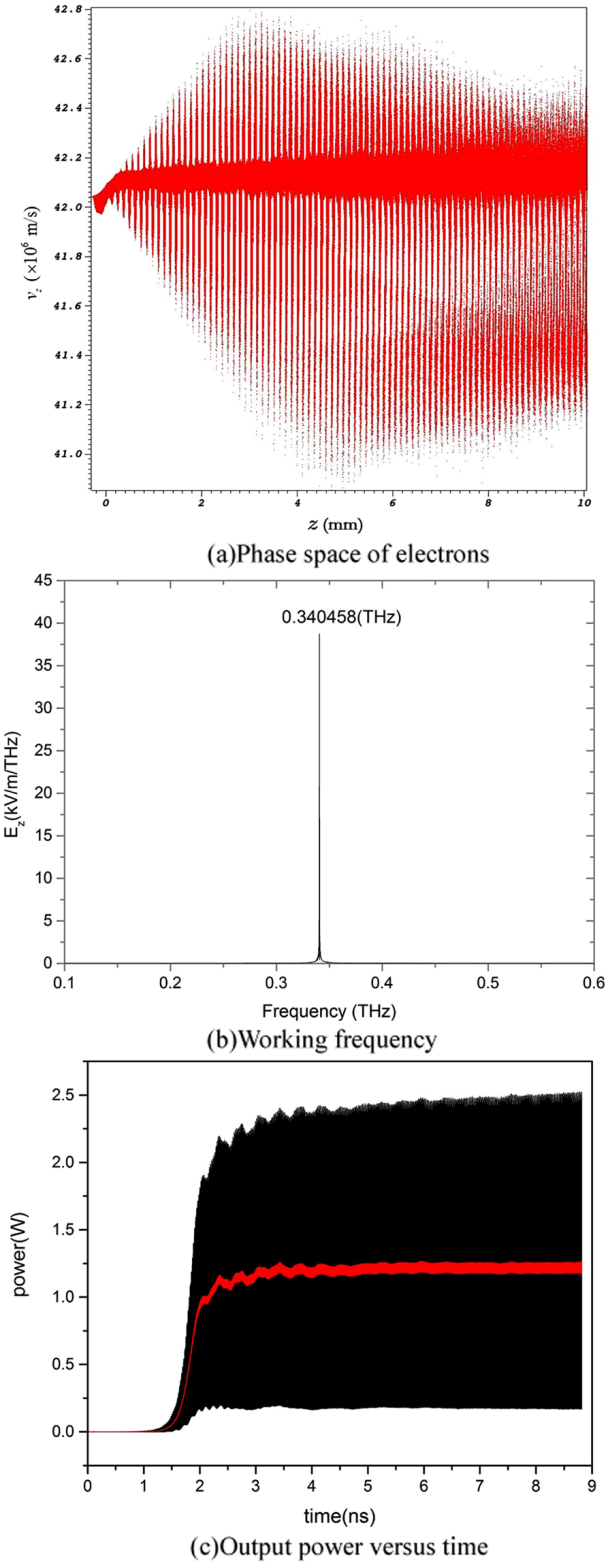
The simulated results of the designed BWO.

To validate the physical design of the BWO, its working characteristics is simulated by using a three dimensional fully electromagnetic particle-in-cell (PIC) code UNIPIC-3D^29,30^, in which the relativistic Newton-Lorentz force equation and Maxwell’s equations are solved on conformal meshes, and the distributing loss on the device wall is included^31^. The cross section of the sheet electron beam with the voltage of 5.0 kV and current of 150 mA is 2.5 mm × 0.14 mm. The electron beam is just 0.01 mm above the grating, so that it can efficiently interact with the −1*st* spatial harmonic of backward wave. Figure 3(a) depicts the electrons’ phase space demonstrating the significant transfer of energy from the electron beam to the terahertz wave, whose working frequency is 0.3406 THz as shown in Fig. 3(b). It is very close to that predicted by the dispersion curves given in Fig. 2(e), indicating that there is no noticeable other modes competition in the BWO. Figure 3(c) shows the output power of the designed BWO, the average output power reaches about 1.2 W.

## Fabrication of the BWO

**Figure 4:**
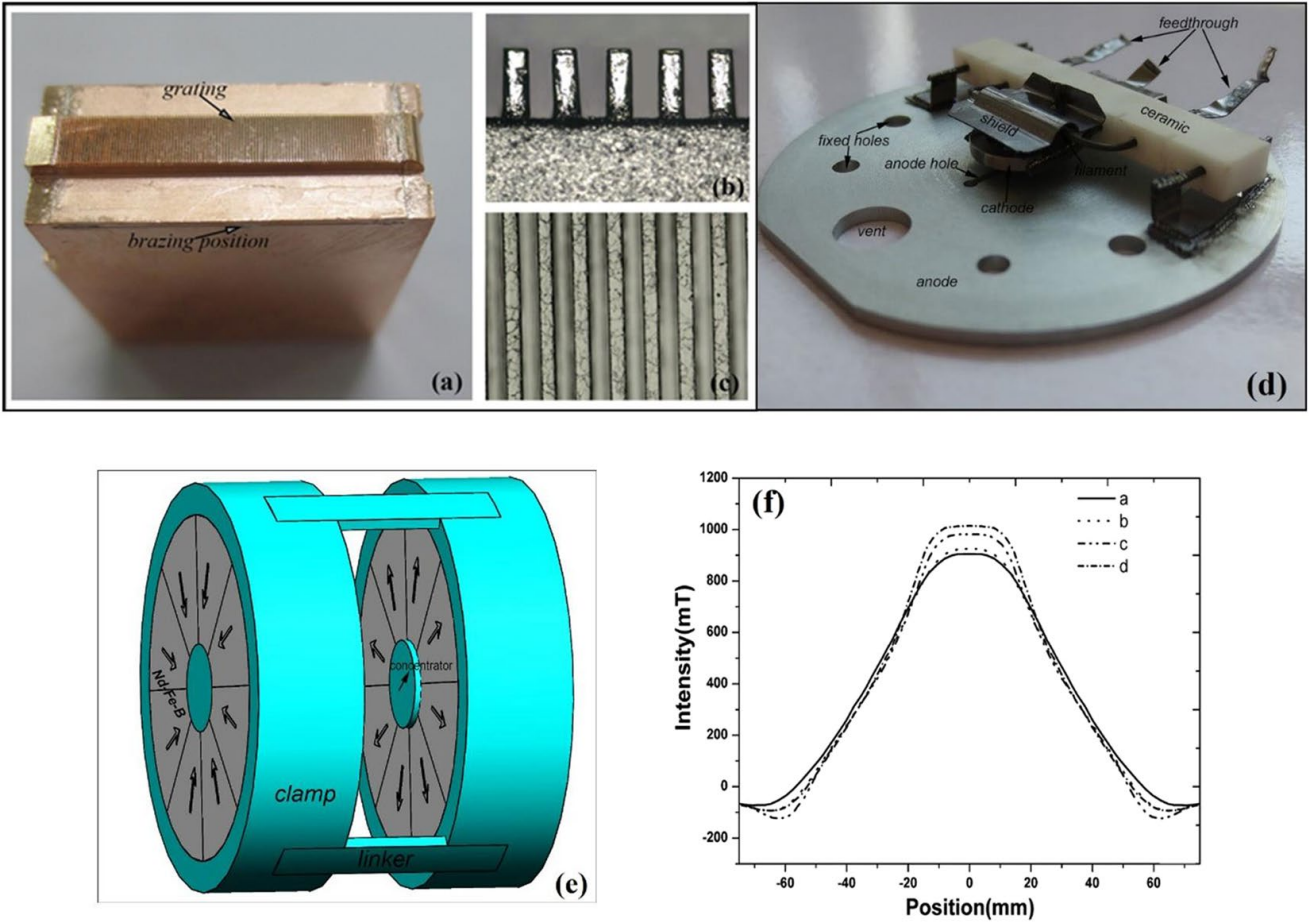
The fabricated components of BWO. (**a**) Whole grating of SWS, (**b**) Side view of SWS, and (**c**) Top view of SWS. (**d**) Photograph of sheet electron gun; and (**e**) The designed MFS. (**f**) Distributions of magnetic field.

The important parts in the planar BWO include the SWS, electron optics system, and magnetic system, etc. In our developed G-band clinotron oscillator^15^, its planar SWSs were fabricated by using the WEDM technology with the processing error less than 5 μm. As the working frequency goes up to the Y-band, this processing error cannot satisfy the requirement of our new device. And hence, we try the UV-LIGA technique to fabricate the planar SWSs^21^. Since the electromagnetic losses increase dramatically at higher frequencies, the copper material is selected for its low millimeter wave loss and high thermal conductivity. Figure 4(a) depicts the completed, all-copper grating fabricated by using the UV-LIGA technique. Figure 4(b,c) are the optical microscopic images of the side view and top view of the grating, showing that the grating structure is uniform and its surface is very smooth. Unlike the WEDM technology, there is no chamfer between the grating and the copper substrate^15^, the grating has vertical side wall, and the average verticality of the grating on the copper substrate is better than 89°. The dimensional error of the fabricated SWS is less than 3 μm, and the surface roughness is less than 100 nm, satisfying the requirement of our Y-band BWO.

As shown in Fig. 4(d), a high-perveance electron gun without beam compression is designed to produce a high quality sheet electron beam. The barium tungsten cathode is used in the electron gun, and the emission slot was cut to 2.5 mm × 0.14 mm. The filament and the cathode are separated, and the filament is exposed to the vacuum system. The temperature of the filament can rise to 1800 °C when turning the DC filament power on. More important, a constant voltage source is connected with the filament and cathode, and there is a heating current to heat the cathode. Through adjusting the filament current and the heating current, the temperature of cathode will be accurately controlled. And hence, the cathode emission current can be controlled accordingly. Though accelerating voltage *U*_a_ can be adjusted to 6 kV, for safety use of the electron gun, Table 1 presents the experimental results of the electron gun versus the *U*_a_ under the conditions of the filament current of 2.6 A and the filament voltage of 3.2 V. It shows that both the emission current *I*_k_ and current density *J* increase with the increase of *U*_a_. The maximum *J* reaches 57 A/cm^2^ at the CW mode without any focusing, which can completely meet the design requirements of the BWO.

**Table 1:** Parameters of the Electron Gun with Sheet Electron Beam.

*U*_a_, V	500	1000	1500	2000	2500	3000	3500	4000	4500	5000
*I*_к_, mА	16	39 ± 1	70 ± 2	103 ± 2	134 ± 3	167 ± 3	173 ± 4	184 ± 5	193 ± 6	200 ± 8
*J*, A/cm^2^	4.57	11.1	20	29.43	38.3	47.7	49.4	52.5	55.1	57.1

To enhance the efficiency of the sheet electron beam, it has to fly a long distance over the comb grating with a small spacing. A precise magnetic focusing system (MFS) is required to make sure the pulsation value of electron beam smaller than the thickness of the layer in which the slow-wave field is concentrated. Both PIC simulation and experimental results indicate that 0.7 T would be the minimal intensity of permanent magnetic field to focus the sheet electron beam in the CW devices. To do so, a kind of MFS with Nd-Fe-B material was developed. Figure 4(e) schematically shows the MFS which has two magnet rings. Each magnet ring contains eight separate magnetized segments which are made of Nd-Fe-B with a residual magnetization of 1.13 T and coercive force of 836 kA/A. The segments in one ring are magnetized from the inner to outer along the radial direction, while the segments in the other ring are magnetized from the outer to inner along the radial direction. There is a columned pure Fe as the magnetic field concentrator to increase the magnetic field induction in the center of each ring, the distance between them is 32 mm, which is a little larger than the thickness of the device. Through optimum design, the thickness, inner and outer diameters of the magnet segments are set to 35 mm, 14 mm and 55 mm, respectively. The measured intensities of the longitudinal component of the magnetic field along the z axis of the magnetic system are presented in Fig. 4(f), where the four curves represent the intensities of magnetic field inside the planes perpendicular to the z axis through the central point a(0,0,0) of the MFS, point b(0,0,4 mm), point c(0,0,7 mm), and point d(0,0,8 mm). It can be easily found that the minimal intensity of magnetic field along the z axis is 0.9 T, the intensity of magnetic field changes little near the central axis, and the radius of uniform magnetic field is larger than 5 mm, which is larger than the cross section of the sheet electron beam. This is good for the long distance transmission of sheet electron beam.

## Experimental Studies on the BWO

**Figure 5:**
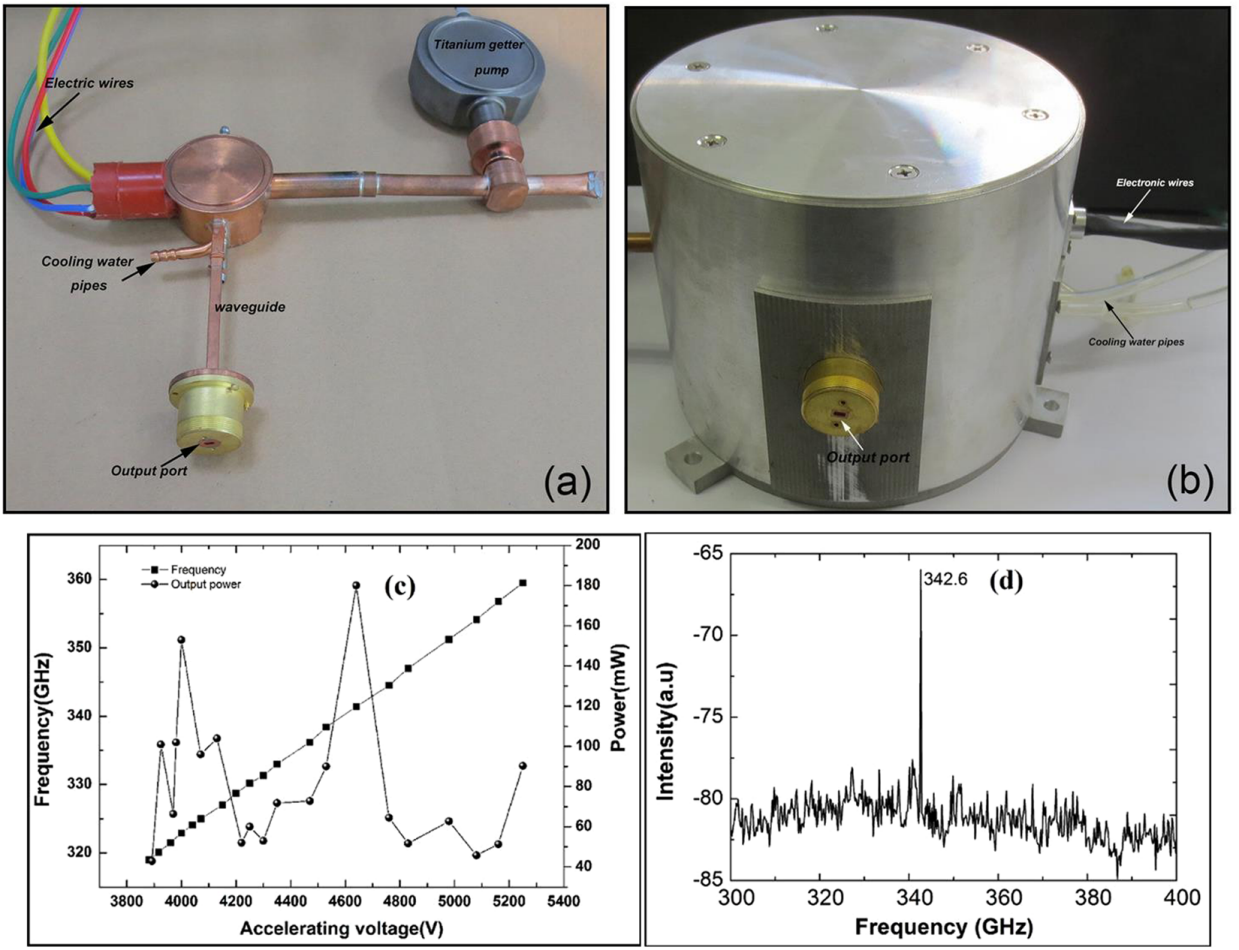
Photography of the assembled BWO after vacuum package and Experimental results. (**a**) Vacuum sealed BWO; (**b**) assembled BWO; (**c**) Output power and frequency vs. accelerating voltage; and (**d**) spectrum of the output signal.

With the high frequency system and electron optics system mentioned above, a kind of BWO was assembled by the electric resistance welding and argon arc welding technology. The tube was evacuated to 2.0 × 10^−7^ Pa by a turbo molecular pump. Figure 5(a) is the vacuum sealed BWO. A titanium getter pump is mounted on the tube, which is used for maintaining high vacuum environment inside the tube and can be cut off after final assemble of the tube. The tube was encased into the assembled MFS shown in Fig. 5(b). The output characteristics of the BWO were investigated with the power meter PM4 and spectrum analyzer developed by the 41st Institute of China Electronics Technology Group Corporation. Experimental results show that the minimum starting current and accelerating voltage are about 64 mA and 3.8 kV, respectively. Under the condition of 135 mA current, the output power and electronic tuning capability with the accelerating voltage are shown in Fig. 5(c). It can be found that the oscillation frequency tuning by changing the accelerating voltage is approximated linearly, the slope is about 29.25 MHz/V, and the oscillation frequency ranges from 0.318 THz to 0.359 THz. The maximum CW output power of the tube achieves 182 mW at the frequency of 0.3426 THz. Figure 5(d) is the output spectrum of the tube at the 4.63 kV. The peak of 0.3426 THz, very close to 0.3414 THz predicted by the dispersion curves given in Fig. 2(e) and 0.3406 THz (Fig. 3(b)) calculated out by the PIC code, is higher than any other signals in the frequency spectrum. The results verify that there is no noticeable other oscillation and mode competition in the tube, which agrees well with the above theoretical analysis. In addition, the 3 dB bandwidth of the output signal is about 4.25 MHz, which shows the designed tubes has good spectral characteristics.

This kind of BWO can operate with stable output power for tens of hours. Under the work conditions of this BWO, a power of about 500 W is dissipated by the SWS. To keep the CW operation, a cooling system is installed in this BWO by using water flowing below the bottom of the SWS (as shown in Fig. 5(b)).

## Conclusions

In conclusion, a kind of BWO is developed in this paper. Through theoretical analysis and simulation, the SWS of the tube is designed as the planar grating structure, which is fabricated by using the UV-LIGA technology with the processing error less than 3 μm. By analyzing the dispersion curve of high frequency structure, distributions of the electric field, and PIC simulation results, the operating mode of the tube is determined as the fundamental mode TM_11_. The high-quality electron optics system can generate a 2.5 mm × 0.14 mm sheet electron beam with maximum current density of 57 A/cm^2^ at the CW mode. Experimental results show that the BWO can operate at the fundamental mode TM_11_ and generate the output power of 182 mW at the frequency of 0.3426 THz with a large frequency tuning range from 0.318 THz to 0.359 THz. The experimental results are obtained under the condition of 135 mA current, corresponding to the current density of 38.6 A/cm^2^, and the life time of the device is about 1000 hours. Like almost all the terahertz VEDs, though the ohmic losses are included in our PIC simulations^31^, the output power is much bigger than the experimental one. The main reasons causing such a significant difference may be the spread of the electron velocities, fabrication error of the structure (especially the SWS), misalignment of the device configuration, and so on. Except the output power, the PIC results are agreed well with those from the analytic design and experiment.

As this kind of BWO has the merits of low voltage, small volume, light weight, room temperature operation and wide-range tuning of the frequency, it will be a promising and effective oscillator in the subterahertz band, and it is anticipated to be widely used for THz imaging and spectroscopy.
